# A New Auditory Multi-Class Brain-Computer Interface Paradigm: Spatial Hearing as an Informative Cue

**DOI:** 10.1371/journal.pone.0009813

**Published:** 2010-04-01

**Authors:** Martijn Schreuder, Benjamin Blankertz, Michael Tangermann

**Affiliations:** 1 Machine Learning Department, Berlin Institute of Technology, Berlin, Germany; 2 Intelligent Data Analysis Group, Fraunhofer FIRST, Berlin, Germany; University of Calgary, Canada

## Abstract

Most P300-based brain-computer interface (BCI) approaches use the visual modality for stimulation. For use with patients suffering from amyotrophic lateral sclerosis (ALS) this might not be the preferable choice because of sight deterioration. Moreover, using a modality different from the visual one minimizes interference with possible visual feedback. Therefore, a multi-class BCI paradigm is proposed that uses spatially distributed, auditory cues. Ten healthy subjects participated in an offline oddball task with the spatial location of the stimuli being a discriminating cue. Experiments were done in free field, with an individual speaker for each location. Different inter-stimulus intervals of 1000 ms, 300 ms and 175 ms were tested. With averaging over multiple repetitions, selection scores went over 90% for most conditions, i.e., in over 90% of the trials the correct location was selected. One subject reached a 100% correct score. Corresponding information transfer rates were high, up to an average score of 17.39 bits/minute for the 175 ms condition (best subject 25.20 bits/minute). When presenting the stimuli through a single speaker, thus effectively canceling the spatial properties of the cue, selection scores went down below 70% for most subjects. We conclude that the proposed spatial auditory paradigm is successful for healthy subjects and shows promising results that may lead to a fast BCI that solely relies on the auditory sense.

## Introduction

Brain-computer interfaces (BCI) are a direct connection between the brain and a computer, without using any of the brain's natural output pathways [1]. Most BCI research is aimed toward developing tools for patients with severe motor disabilities and paralyzes, patients suffering from amyotrophic lateral sclerosis (ALS) specifically. This group of potential users could particularly benefit from BCI technology, since output pathways that are normally employed by the brain can no longer be used. Completely locked-in syndrome (CLIS) patients have lost all volitional control over their muscles, including eye-muscles, and are therefore out of reach for conventional augmentation devices based on rudimentary muscle control. BCI might be one of the last options for communication for these patients.

BCI research over the last decades has explored a large variety of possible configurations for such a BCI. Among these are the choice for measuring method, physiological brain feature, analysis method and modality of interaction. So far, the primary choice of interaction modality has been vision. Most current BCI systems rely to some extent on the ability of the subject to control the eyes. However, the patients' inability to direct gaze, adjust focus or perform eye-blinks may proof the use of the visual modality in BCI application to be difficult. Therefore, other modalities are now being explored such as audition [2]–[10] and touch [11]–[14] in order to make BCI independent of vision. Moreover, when using such alternative methods for patients with residual vision, the visual modality could be used exclusively for feedback, thereby preventing interaction between feedback and stimulation.

Current auditory BCI systems mostly result in a binary decision. Binary decisions contain lower information content than multi class decisions. Although for some tasks a multi class BCI is the best choice, it is difficult to cope with multiple options in the auditory domain. In the current research we look for alternative ways of stimulus presentation that will allow for a multi class auditory BCI. We hypothesize that by adding spatial information to the cues, subjects will be able to discriminate a larger number of classes. If classification of the P300 deflection in response to this spatial information is possible, it introduces a new means of creating a truly auditory BCI. Such a setup would be flexible in the number of classes used and could potentially increase the speed of auditory BCI.

### Auditory BCI

Hill et al. [2] used event-related potentials (ERP) that are triggered by auditory stimuli for a binary BCI. They presented two sequences of deviant (target) and standard (non-target) tones to the subject. Both ears received a sequence with a different inter-stimulus interval (ISI) at the same time. The subject's task was to focus on either one of the streams by counting the number of targets in that stream. The time samples for left and right non-target tones were taken from the same four seconds of EEG, averaged and subsequently concatenated. Because of the different ISI, ERP in response to the left channel would average out on the right channel samples and vice versa. This concatenated feature was used for classification. Although the classification rate varied widely between different subjects, their results are promising for the use of auditory ERP as a feature for BCI.

A similar approach was recently reported in [7]. They used the human capacity to segregate audio streams to create a binary BCI. Two different oddball audio streams were presented to the subject's right ear. When the ISI of such streams is short, the subject naturally segregates these into independent streams. For classification, the ERPs to both streams were classified and the target stream was determined by voting over multiple presentations. Although it is a binary BCI, they argue that it could be extended by adding more streams and thus increase the number of classes. Unfortunately, they used all data for training and testing for their reported results, rather than using a cross-validation method.

Another attempt to create a BCI that is independent of vision used auditory feedback to inform subjects on their sensory motor rhythm (SMR) [3]. By adjusting their SMR, subjects were able to make a binary choice. Although initial performance for most subjects was better with visual feedback, this difference decreased with learning. Thus, as they conclude, auditory feedback can effectively be used for a BCI based on SMR.

Similarly, Hinterberger et al. [8] used auditory feedback to inform subjects on their control of the slow cortical potential (SCP). Although two subjects reached the 70% accuracy score that is assumed to be minimal for useful BCI operation [15], they generally performed worse than subjects with visual feedback. Furthermore, a BCI based on SCPs typically requires several sessions of training until an acceptable level of BCI control can be obtained.

Even different ways of using the auditory modality have been investigated, such as frequency tagging. When a high frequency tone with a low frequency envelope is presented to a subject, the frequency of the envelope has been found to resonate in the EEG signal. The extend of this resonating can to some level be influenced by selective attention [6]. This envelope could also be constructed as pseudo-random noise, which allows for the use of multiple streams [9].

### P300 response

The P300 feature of the human brain is a well-described positive deflection of the ongoing EEG signal [16], [17] with a latency of 300+ ms to an event. In most people it is present without training in response to an attended rare event. The task that is generally used for eliciting a P300 wave is the oddball paradigm, where an attended target stimulus is infrequently presented between non-target stimuli. The attended stimulus elicits a P300 response in the brain, which generally has the largest amplitude at the midline Pz electrode and parietal regions [18]. The P300 was shown to be greater with larger target-to-target intervals [19]. Stimulus order in an oddball paradigm should be random to prevent expectation of the target stimulus.

In the setting of BCI, the short latency of the P300 allows for fast communication speeds. Stimuli can even be presented at a pace faster than the actual timeline of the P300, thereby further increasing the efficiency. This has primarily been done in the visual P300 speller [4], [20]–[22]. Because the P300 response is elicited by an external stimulus, operation speed is dictated by the rate of presentation of these stimuli. This is referred to as synchronous operation mode.

Although it is well established that the P300 component only requires covert attention, it turns out that the performance of visual P300 BCIs degrades if the target stimulus is not overtly fixated [23]. Overt fixation is not a relevant factor in the auditory domain, but the P300 was shown to be stronger for attended stimuli in auditory mode [4]. They showed in a four stimulus oddball task that the P300 response is present when the target stimulus is presented visually, auditory and in a combination of both. Similarly, the P300 response was reported to be attention dependent when tactile stimuli are used [14].

The visual P300 response has been used for BCI [24], [25], in particular for creating a speller application [4], [20]–[22]. In the latter, a matrix of characters is presented and the rows and columns light up in random sequence. The subject attends to the character he/she wants to select by counting the number of illuminations. When the row or column containing the character lights up it elicits a P300 wave, which can be detected from the EEG. Thus, the row and column that give a P300 response define the character that is to be selected. Nijboer et al. [22] showed that this paradigm can be successfully used by ALS patients.

A similar selection process has been devised for the auditory modality [5]. The matrix was still shown for reference purposes, but the columns and rows did no longer flash. Instead, they were marked by a spoken number that was presented to the subject. The subject no longer attended visually, but was instructed to attend to the spoken number that identified the character. They compared the performance with the visual speller. Although the visual speller had significantly better results, satisfiable results were found in the auditory condition as well, with performance reaching up to 100% for one subject. However, auditory stimulation with spoken numbers is time consuming, and selection of a letter could take as long as 3.6 minutes when using multiple iterations.

In a recent publication [10] the rows and columns were sequentially represented by six natural sounds. The subject would be visually informed on which sound corresponded to a row or column; a mapping that most subjects could learn within 2 sessions. Subjects were divided in two groups, one group received only the auditory stimuli whereas the second group received concurrent auditory and visual stimulation. For the second group, the number of trials with visual stimulation would gradually be reduced. After 11 sessions, both groups received only auditory stimulation. Although accuracy in session one was lowest for the auditory only group, their performance on the 11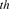 session had increased to a level comparable to that from the combined stimulation group.

The oddball principle has also been used for action selection through spoken word stimuli [4]. In an oddball paradigm setup they showed that short spoken words lead to a P300 response when attended to. They used simple words (‘*YES*’, ‘*NO*’, ‘*PASS*’, ‘*END*’) as target/non-target combinations. Although this leads to a less distinct P300 than in the visual modality, it could be classified by averaging over subtrials.

### Spatial hearing

Localization of sounds in space is one of the processes that our brain does without mental effort. For an extensive review on spatial hearing in humans see [26]. Several behavioral studies have shown the ability of human listeners to distinguish sounds in space [27]–[29]. Several of these studies further showed that when subjects focus on a particular direction, their attentional resources appear to be distributed in a gradient, with decreasing alertness when moving away from the attended direction [28], [29].

Although most oddball experiments employ a cue with a difference in pitch, amplitude or length of the stimulus sound, other properties of sound have been investigated. One such property is the spatial location of the stimulus. In their study, [29] essentially presented seven oddball paradigms to the subject. An array of seven speakers (with 9 distance between them) presented non-targets and targets on all directions in random order. Subjects were asked to attend left, front or right and only targets coming from the attended direction elicited a P300 reliably. In this case, the spatial location is not used to separate non-targets from targets but rather to separate different streams. A more recent study did use the spatial separation (albeit virtually through stereo headphones) to set aside the frequent non-targets (0, straight ahead) from the infrequent targets (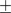
30 and 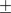
90) [30]. However, because the subjects were engaged in a passive listening task and in the meanwhile watched a movie, no P300 responses were elicited. Rather, the focus was on the early mismatch negativity potential. It does show, that spatial location can be a cue determining factor. A similar experiment was performed in free-field with only 10 spatial separation [31].

An oddball paradigm purely based on spatial location has been used in [32], but merely as a training for detecting stimuli from different locations in a later task. No behavioral- or neurophysiological data for this condition is reported.

## Methods

### Ethics statement

Procedures were positively evaluated by the Ethics Committee of the Charité University Hospital (number EA4/073/09). All subjects provided verbal informed consent and subsequent analysis and presentation of data was anonymized.

### Participants

Two sets of experiments were performed. The first set (physiological experiments) included seven healthy volunteers (five male, mean age 29.1 years, range 25–34 years) and was used for validation of the setup and assessment of the physiological response. All subjects were volunteering group members and had some previous experience with BCI, mainly based on imagined movement tasks. The second set (BCI experiments) included five healthy volunteers (three male, mean age 32.4 years, range 22–55 years), out of which two were paid subjects with no previous experience in BCI. They were compensated for their time with eight euro per hour. Of the other three volunteering group members, two also participated in the first round.

Subjects reported to be free of neurological symptoms and to have normal hearing, although two subjects (VPip and VPig) reported having difficulty with spatial localization of sounds in natural situations and subject VPzq reported a high-pitched tinnitus in the right ear.

### Task, procedure, and design

**Figure 1:**
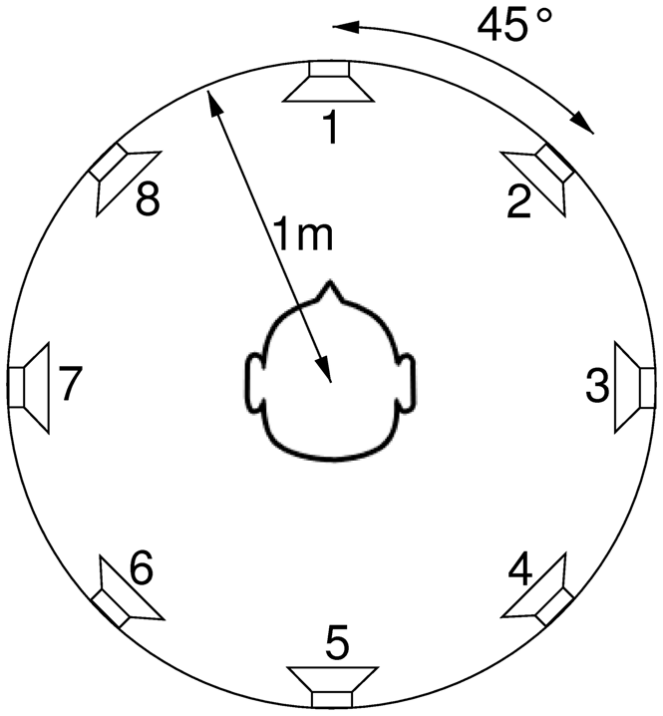
The experimental setup. For the physiological experiments, all eight speakers were used. For the BCI experiments, only the front semi-circle was used (speakers 1,2,3,7, and 8).

Subjects sat in a comfortable chair, facing a screen with fixation cross. They were surrounded by eight speakers at ear height. The speakers were spaced evenly with 45 angle between them, at approximately one meter distance from the subject's ears (see Figure 1). Speakers were calibrated to a common stimulus intensity of 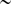
58 dB. At the start of each recording session, subjects were asked to judge the subjective equality of the loudness from all directions and alter these if necessary. The room was neither electromagnetically shielded, nor were any sound attenuation precautions taken. All experiments consisted of an auditory oddball task that varied to some degree. Before the experiments, subjects were asked to minimize eye movements and other muscle contractions during the experiment. Stimuli were generated in Matlab and presented using the PsychToolbox [33]. A multichannel, low-latency firewire soundcard from M-Audio (M-Audio Firewire 410) was used to individually control the low-budget, off-the-shelf computer speakers.

#### Physiological experiments

First, experiments were performed to assess the physiological response to the setup. All eight speakers were used and the stimuli consisted of 75 ms bandpass filtered white noise (150–8000 Hz) with 3 ms rise and fall. The stimulus for all speakers was the same, making spatial location the only discriminating cue. Any one of the eight directions could be a target (probability 12.5%), leaving the others as non-targets (probability 87.5%). Therefore, this can be considered a classic oddball paradigm. The target direction was indicated prior to each block, both visually on the screen and by presenting the stimulus from that location.

In condition C1000, one trial consisted of 80 subtrials, ten for each individual location. We recorded 32 of such trials, making a total of 2560 subtrials. Inter-stimulus interval (ISI) was set to one second with a latency jitter (mean 25 ms, SD 14.4 ms). Subjects were asked to mentally keep track of the amount of target stimulations.

In order to have an indication of the subjects recognition performance, a second condition (condition Cr) was introduced. Instead of mental counting, subjects were asked to respond by key press each time the target direction was stimulated. To allow for a response, the ISI was set to two seconds with the same latency jitter. Between 576 and 768 subtrials per subject were recorded. Blocks of both conditions were mixed to prevent time biases.

If necessary, an initial round of stimuli was given before recording to familiarize the subject with the stimuli. Presentation order was pseudo random with the restriction that all eight directions were stimulated in one block before continuing to the next block.

#### BCI experiments

For the BCI experiments, the paradigm was altered in several ways based on findings from the physiological experiments. First, the amount of speakers was reduced to the frontal five to make the task easier. Thus, the target was presented with 20% probability and non-targets with 80% probability. It has been shown that this is rare enough to produce a P300 response [4]. All five speakers were given a unique, complex 40 ms stimulus, build from band-pass filtered white noise with a tone overlay (see Table 1 and File S1). The discriminating cues were now both the physical properties and the spatial location of the stimulus. Latency jitter was omitted. In order to explore the boundaries of the paradigm, three different conditions were tested.

**Table 1:** Cue properties in BCI experimental round.

Direction	Nr	Lower bound (Hz)	Upper bound (Hz)	Tone (Hz)
Left	7	320	2500	440 (a)
Front-left	8	416	3250	494 (b)
Front	1	540	4225	554 (cis)
Front-right	2	703	5493	622 (dis)
Right	3	914	7140	699 (f)

*Nr* refers to the speakers labels given in Figure 1. *Lower*- and *Upper bound* are the boundary frequencies for the band pass filter that is applied to the white noise. *Tone* is the fundamental frequency of the tone overlay. Seven harmonics were used, with decaying amplitude. Tone frequencies are chosen to have a full note in between adjacent stimuli.

The first two conditions differed in their ISI (300 ms for condition C300, 175 ms for condition C175). A trial consisted of 75 subtrials, 15 for each location. For condition C300, we recorded 50 of such trials, making a total of 3750 subtrials. For condition C175, 40 such trials were recorded making a total of 3000 subtrials.

The third condition (C300s) also had a 300 ms ISI. However, all stimuli were now presented through a single speaker (front), thereby leaving the pitch properties of the stimulus the only discriminating cue. Only 20 trials of 75 subtrials were recorded for this condition, making a total of 1500 subtrials. Blocks of the three conditions were mixed to prevent time biases.

Stimuli order now had the extra constraint that there were at least two other directions between presentations of the same direction, to prevent too much overlap of target time frames. If necessary, an initial round of stimuli was given before recording to familiarize the subject with the stimuli.

### Artifact rejection

For artifact rejection, a simple threshold method was used. The epoched data was first detrended to avoid slow drifts from reaching the threshold. Then, subtrials with a deflection greater than 70 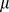
V over the ocular channels, compared to baseline, were marked as artifacts. These subtrials were then rejected from the original data and excluded from further analysis. This method excludes mainly eye artifacts.

### Data acquisition

EEG was recorded monopolarly using a varying number of Ag/AgCl electrodes. Channels were referenced to the nose. Electrooculogram (EOG) was recorded with two bipolar channels over the eyes. The signals were amplified using a Brain Products 128-channel amplifier, sampled at 1 kHz and filtered by an analog bandpass filter between 0.1 and 250 Hz before being digitized and stored for offline analysis. Further analyzes were done in Matlab (The Mathworks, Version 7.4).

For visual inspection, the raw data was low-pass filtered with an order 8 Chebyshev II filter (30 Hz pass-frequency, 42 Hz stop-frequency, 50 dB damping) to remove obvious 50 Hz artifacts from external sources. The filter was applied to the data both forward and backward to minimize phase-shifts. After filtering, the data was down sampled to 100 Hz and epoched between -150 ms and 800 ms relative to stimulus onset, using the first 150 ms as baseline. Artifacts were disregarded by the simple method described before. P300 latencies and amplitudes were calculated on the 1000 Hz data directly, using the same filters as described above.

For classification purposes the same filter was used before down sampling to 100 Hz. However, the filter was applied causally (only forward) to ensure portability to the online setting, where no future samples are available. Data was epoched in the same way as described above. The same artifact rejection method was used.

### Analysis

We use a measure derived from the receiver operating characteristic (ROC, [34]) to quantify the separability of two one-dimensional distributions. While ROC curves and derived measures are often used to characterize the performance of classifiers [35], they can as well be used to quantify the discriminability of feature distributions. The advantage over methods like Fisher score [36], [37], Student's *t*-statistic [37], [38] or pointwise biserial correlation coefficient, is that it does not rely on the assumption that the distributions are Gaussian.

The ROC curve of perfectly mixed distributions is (approximately) the diagonal line (no-discrimination line), and the ROC curve of perfectly separated distributions is a right angle going from (0,0) either through (1,0) or through (0,1) to (1,1). As separability index, we use the signed area (as in the definite integral) between the ROC curve and the no-discrimination line multiplied by two, such that the range of this scoring is between −1 and 1. So, if all values of class 1 are strictly larger than the maximum value of class 2, the ROC-separability-index is 1; if all values of class 1 are smaller than the minimum value of class 2 the index is −1. Accordingly, this separability index is similar to the point biserial correlation coefficient, but does not rely on the assumption that the classes obey Gaussian distributions.

For condition C1000, grand averages were computed for the channels with the highest (P300, interval 300–650 ms) and lowest (N2, interval 100–300 ms) signed ROC value, as well as scalp topographies for the intervals where these peak ROC values were found. Furthermore, response times and errors from condition Cr were computed. For the BCI experiments grand averages were computed for the channel with the highest signed ROC values only. Scalp topographies were only computed for the C175 condition, again in the interval where the high peak ROC values were found.

Due to the temporal aspect of the P300 response, the EEG trace itself was used as a feature for classification. The 20 channels that accounted for most of the difference between the two classes were automatically selected within each fold of the crossvalidation. For this, the ROC values were calculated for each channel and sample. The 10 channels with the highest positive ROC peak and those 10 with the lowest negative ROC peak were used. Data from these channels were decimated by taking the mean of five samples, effectively reducing the data to 16 post-baseline samples per channel. Samples from all 20 channels were then concatenated to form a 320 dimensional feature vector. The feature vector of the training set was normalized to zero mean and unit variance for every dimension independently and the normalization vector stored to normalize subtrials of the test set.

Classification was done using the Fisher Discriminant (FD) algorithm. Due to the dimensionality of the features (320 dimensions), some form of regularization was advisable. Here, a shrinkage method which counterbalances the systematic error in the calculation of the empirical covariance matrix was used [39]. A ten-fold cross validation was performed with ten chronologically sampled partitions. Each partition functioned once as test set with the other nine partitions as training set.

Two types of classification scores can be distinguished: classification- and selection score [5]. Here, classification score refers to the binary classification. It is defined as the percentage of subtrials that is correctly scored to be a target or non-target. The selection accuracy denotes the percentage of trials in which the target direction is correctly designated.

Datasets from the BCI experiments contained four times more non-target stimuli than targets. Although the classification task is essentially binary, chance level for classification is 80%, which could potentially be obtained by simply assigning all samples to the non-target group. Therefore, the number of misclassified targets was checked.

### Multi class selection

After the cross validation, the classifier output was used to determine the outcome of the multi-class paradigm, i.e., to estimate the target direction. Taking a set of consecutive subtrials, one for each direction, the subtrial with the most negative classifier output was designated the target. One such set is referred to as an iteration.

To increase sensitivity, outcomes of multiple subtrials for the same direction (within one trial) can be averaged. This way, the influence of single subtrials is decreased and the selection score can be more robust. One possibility is to average the raw subtrial timeseries for each direction and classify these as a single subtrial. Another option is to classify each original subtrial individually and average over the classifier scores. We took the latter approach, as early results showed better performance for this method.

Artifacts were rejected and as a result classification scores for some directions were missing. Because only the remaining valid subtrials were considered, the averaging for some directions was done over less than the stated number of iterations. This is a realistic approach for future online settings, where artifacts may occur at any time, even in patients. Various amounts of iterations were considered to evaluate the influence on the outcome.

### Information Transfer Rate

The amount of information carried by every selection can be quantified by the information-transfer rate [40, ITR], defined as:
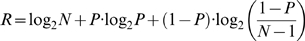
(1)

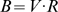
(2)where *R* is the bits/selection and *B* the bits/minute. *N* is the number of classes, *P* the classifier accuracy and *V* is the classification speed in selections/minute. In our case, when using multiple iterations, *P* is the selection accuracy. From this it is clear that even though the selection accuracy may increase when using more iterations, the ITR may stay the same or even decrease because a selection takes more time, i.e., V increases.

Speed is not the only factor that decides on the usability of a BCI, accuracy is equally important. Some applications may require high speed and can deal with lower accuracy (for instance in gaming), whereas other applications need an accuracy that approaches 100% at the cost of speed (such as operating a wheelchair). A higher accuracy is generally obtained by using more trials, i.e., increase the number of iterations. In order to compare our system on both levels, we report two ITR measures. The first, ‘Max ITR 70%’, refers to the maximum ITR that can be obtained when only taking into account the amount of iterations that result in a selection score of 70% or more. Although this is not necessary the highest ITR, we do not regard selection scores lower than 70% as useful because this would require a large number of error corrections; it would not give the subject a sense of control. The second measure, ‘Max ITR 90%’, is based on only those numbers of iteration that result in a selection score of 90% or higher. In general, this means using more iterations and possibly a decrease of the ITR. However, the increased selection accuracy and sense of control may be favorable for some applications.

Rejected trials were still considered during calculation of the ITR to prevent an artificially small approximation of *V*.

## Results

### Artifact rejection

For subject VPiz more than half of the subtrials had to be excluded because of artifacts. In condition C1000, on average about 20% of all subtrials were excluded from analysis (range 5.94%–58.48%). This is about twice as high as the average rejection rate for the other conditions. A possible explanation is the long ISI. As with longer ISI the total length of the trial increases, eye blinks may become unavoidable after some time. Number of rejected trials for all conditions can be found in Table 2.

**Table 2:** Rejection rates for all conditions.

Subject	C1000	C300	C175	C300s
VPiz	1497 (58.48)	- -	- -	- -
VPip	276 (10.78)	- -	- -	- -
VPig	152 (5.94)	- -	- -	- -
VPjf	624 (24.38)	- -	- -	- -
VPjb	525 (20.51)	- -	- -	- -
VPja	160 (6.25)	242 (6.45)	205 (6.83)	125 (8.33)
VPzq	340 (13.28)	184 (4.91)	107 (3.57)	104 (6.93)
VPkh	- -	1104 (29.44)	1037 (34.57)	302 (20.13)
VPkj	- -	113 (3.01)	42 (1.40)	71 (4.73)
VPjq	- -	211 (5.63)	87 (2.90)	42 (2.80)
**Average**	510.6 (19.94)	370.8 (9.89)	295.6 (9.85)	128.8 (8.59)

Using the simple artifact rejection method explained before, between 1.40% and 58.48% of the trials were rejected as artifacts. The average rejection rate for condition C1000 is almost twice as high as that for the other conditions. Possibly this is due to the longer ISI, which results in a longer overall trial. Eye blinking may be unavoidable in this case.

### Physiological response

**Figure 2:**
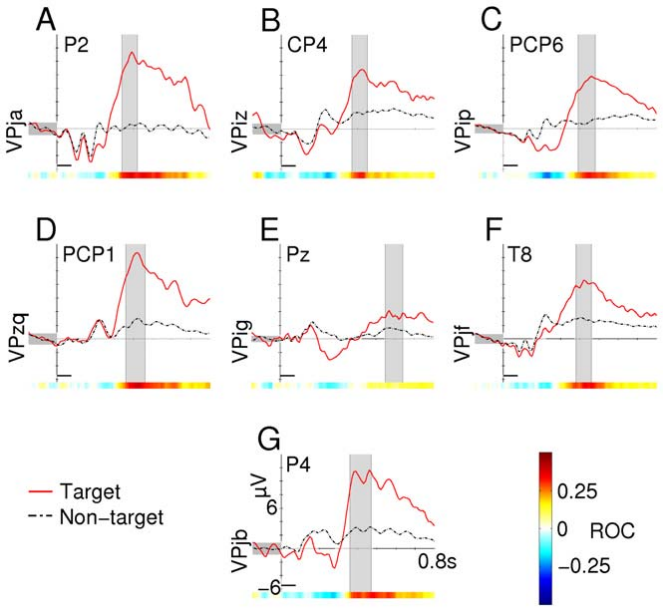
Averaged positive waveforms (condition C1000). Only the channel with the highest positive ROC value between 300 and 650 ms is presented here. The shaded interval indicates the area where this highest ROC value was found. Intervals were handpicked. Scalp topographies in Figure 3 are taken from this interval. Horizontal black bars mark the time of stimulus presentation.

**Figure 3:**
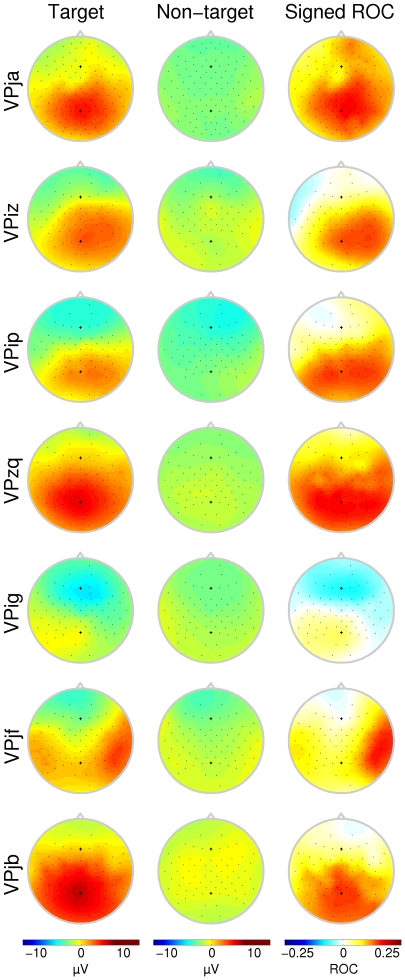
Scalp topographies for the P300 interval (condition C1000). Scalp topographies indicate the average potential over the interval marked in Figure 2. ROC plots do not necessarily indicate the magnitude of the difference between the two curves, but rather the significance of that difference. For most subjects this is concentrated over the parietal area. Each row corresponds to a different subject. Note that not all subjects have the same number of electrodes available.

Averaged ERP responses and scalp topographies for all subjects in condition C1000 can be found in Figure 2 and Figure 3, respectively. For the ERP plots, the channel with the highest positive ROC value between 300 and 650 ms post-stimulus is shown for each subject (Figure 2). Plots show a single target and non-target line; data from all directions is averaged together. The same is done for channels with the largest negative ROC value between 100 and 300 ms. Latency and amplitude of the P300 response can be found in Table 3.

**Table 3:** P300 waveform characteristics.

Subject	Peak latency (ms)	Peak amplitude ( 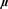 V)	Channel
VPja	385	11.56	Pz
VPiz	411	8.68	Pz
VPip	454	8.14	PO1
VPzq	418	12.48	PCP1
VPig	564	4.53	P01
VPjf	415	10.02	CCP8
VPjb	459	12.02	PO2
**Average**	443.71	9.63	**-**

The peak is defined as the point with the maximum potential in the target class in the interval between 300 ms and 650 ms. Data are taken from the channel indicated. For every subject the channel with the highest positive *ROC* value within the time interval was chosen. This is not necessarily the channel with the largest peak, but the channel with the most significant difference between the responses to targets and non-targets.

**Figure 4:**
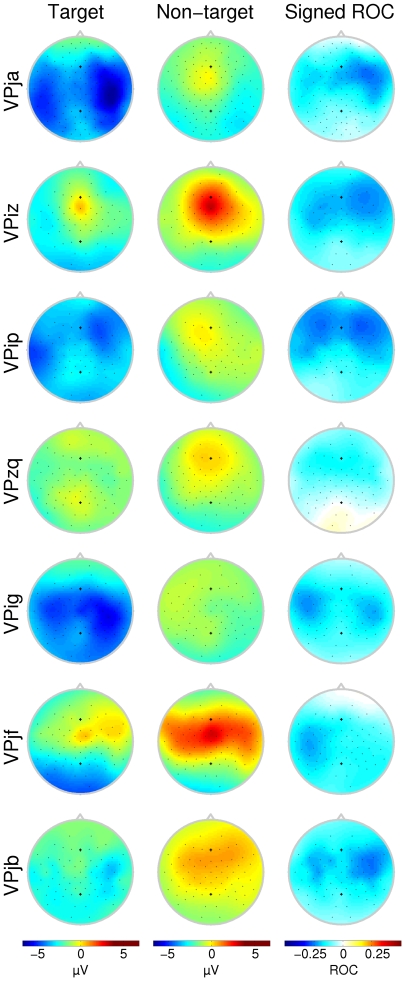
Scalp topographies for the negative deflections (condition C1000). Scalp topographies indicate the average potential over the interval marked in Figure 5. ROC plots do not necessarily indicate the magnitude of the difference between the two curves, but rather the significance of that difference. For most subjects this is concentrated over the frontal and temporal area. Each row corresponds to a different subject. Note that not all subjects have the same number of electrodes available.

In condition C1000, all but subjects VPig and VPjf had a typical P300 response concentrated over the parietal areas with an average latency of 425.4 ms. Although the channel with the highest ROC value was not necessarily directly over the vertex, scalp topographies in Figure 3 show that for these 5 subjects, the distribution of the positive deflection was concentrated around the *Pz* electrode. Subjects VPig and VPjf had an exceptional scalp topography. Subject VPjf showed a typical P300 response in the timeseries, however, distribution of channels with high ROC values was lateralized to the right (see Figure 3, row 6). Subject VPig showed a slight P300 effect over the parietal area, with relative large latency (564 ms). Although the positive ROC value for this subject was very low and the response error was high (see Table 4), selection scores were still over 90% (not presented here). This is possibly due to a large negative class difference found over the frontal electrodes (see Figure 4, row 6).

**Table 4:** Subject performances for the key response task.

Subject	RT [ms]	Hits	False alarms	Misses	Error
VPja	456 (128)	72	3	0	4%
VPiz	479 (148)	71	0	1	1.4%
VPip	507 (174)	72	1	0	1.4%
VPzq	360 (82)	71	5	1	7.8%
VPig	612 (219)	88	17	8	22.1%
VPjf	360 (131)	96	4	0	4%
VPjb	450 (113)	95	3	1	4%
**Average**	460.6 (142.1)	-	-	-	6.4%

Because the majority of stimuli is not a target, true negatives (no response to non-target) are not reported and also not counted for the error score (see equation 3). The total number of targets is equal to the sum of hits and misses. *RT* is the average reaction time from correct responses, with standard deviation in parentheses.

#### Negative deflections

**Figure 5:**
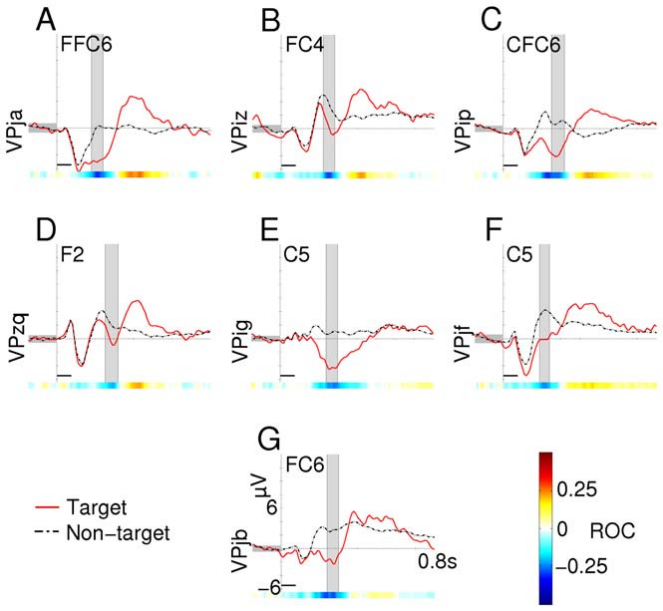
Averaged negative waveforms (condition C1000). Only the channel with the largest negative ROC value between 100 and 300 ms is presented here. The shaded interval indicates the area where this largest negative ROC value was found. Intervals were handpicked. Scalp topographies in Figure 4 are taken from this interval. Horizontal black bars mark the time of stimulus presentation.

Attentional effort not only influences the positive P300 response, but has also been shown to alter the negative deflections prior to the P300 response [41]. Although distinct N1 and N2 components were not always found (see Figure 5), there was a negative class difference over the frontal areas and those electrodes over the auditory cortex for most subjects. Subject VPig, who had no clear P300 response, did show a pronounced attention dependent negativity over both auditory cortices (see Figure 4, row 5). On the other hand, subject VPzq, who had a very typical P300 response with high ROC value (Figure 3, row 4) hardly showed any attentional influence on the negative peaks (Figure 4, row 4). One other remarkable observation is the localization of the negative ROC values for subject VPjf. Where the attentional effect on the positive P300 response was localized over the right central area, the largest negative ROC values were found over the same area on the opposite hemisphere.

#### BCI experiments

**Figure 6:**
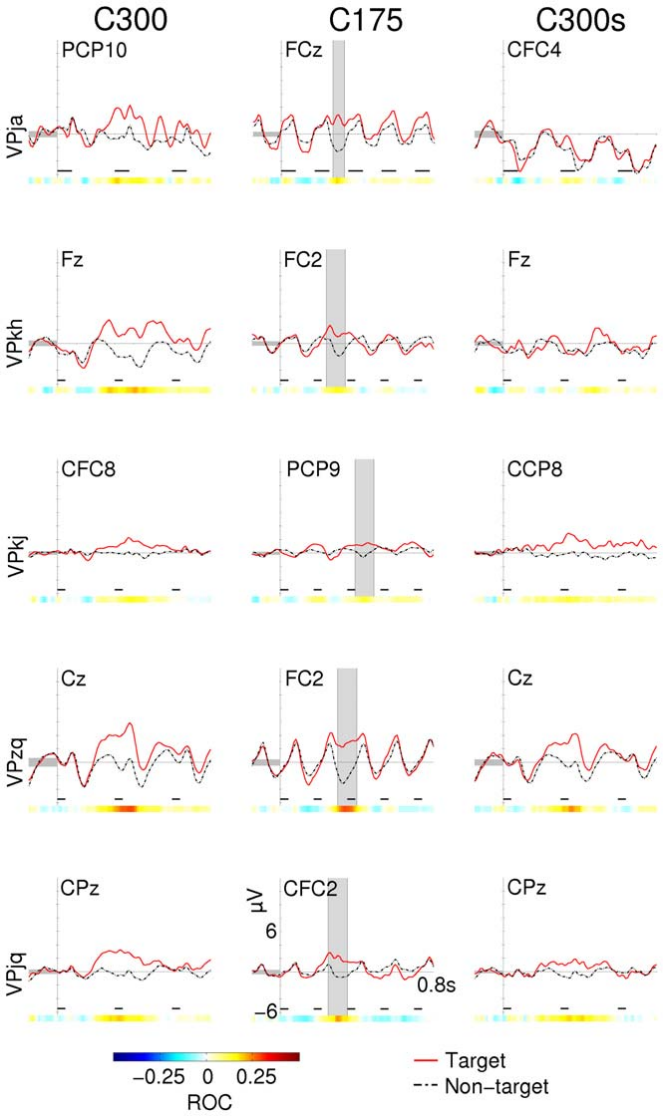
Averaged waveforms of all subjects and conditions from the second experimental round. Only the channel with the highest ROC value between classes is presented here. All ERPs in the left column come from condition C300. The middle column represents condition C175 and images in the right column are taken from condition C300s. Every row represents a subject. The shaded area in condition C175 marks the high ROC interval that is used for scalp topographies in Figure 7. Horizontal black bars mark the time of stimulus presentation.

**Figure 7:**
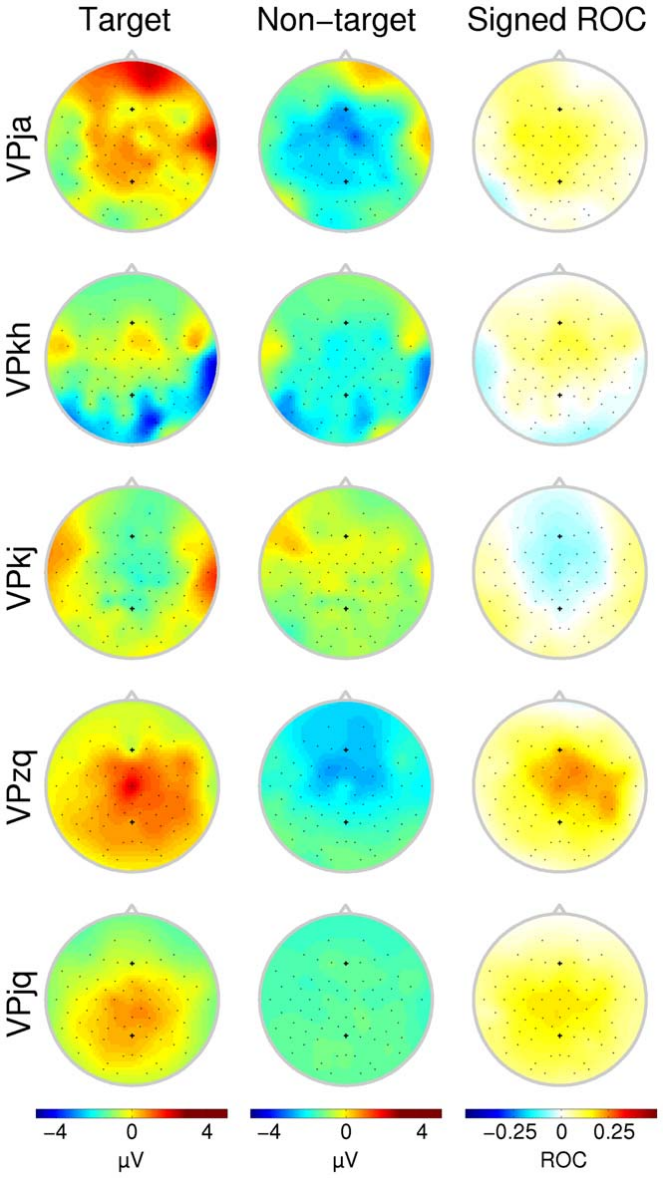
Scalp topographies for the P300 interval (condition C175). Scalp topographies indicate the average potential over the interval marked in the second column of Figure 6. ROC plots do not necessarily indicate the magnitude of the difference between the two curves, but rather the significance of that difference. The area where the high ROC values are concentrated has shifted to the frontal area as compared to Figure 3. Each row corresponds to a different subject. Note that not all subjects have the same number of electrodes available.

**Figure 8:**
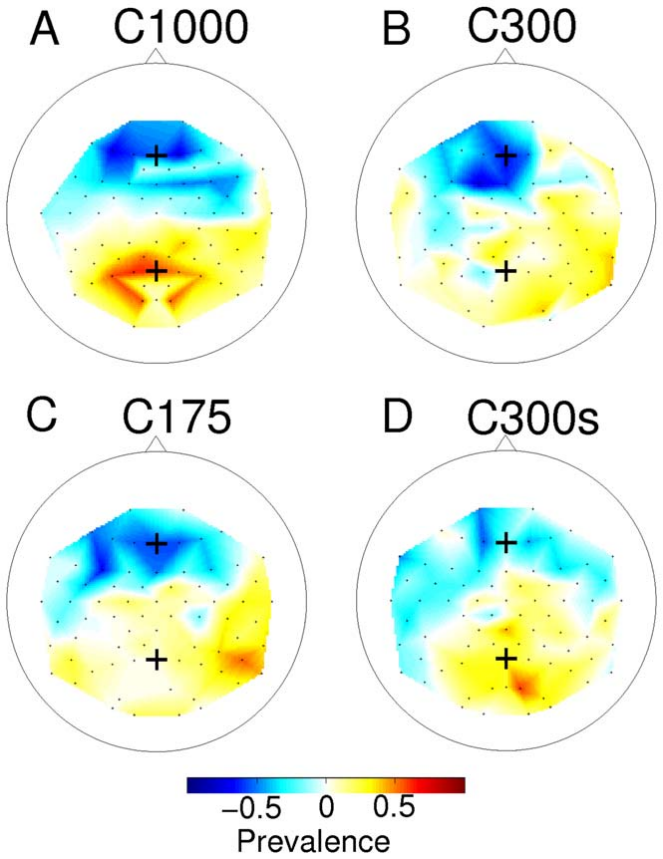
Distribution of channels selected for classification in various conditions over all subjects. Scalp topographies indicate the prevalence of selection of different channels during the cross validation steps averaged over all subjects of a particular condition. Negative values indicate channels selected for negative ROC values, positive values indicate channels selected for positive ROC values. Values have been normalized to the maximum possible occurrences (nr subjects x nr of crossvalidation folds). The frontal cross indicates the *Fz* channel, the posterior cross indicates the *Pz* channel. Channels with negative ROC values are consistently selected from the frontal regions. For condition C1000, the channels with positive ROC values are concentrated over the parietal- and occipital areas, whereas for the faster conditions these are more diffuse.

For comparison, the ERP responses for all subjects and conditions of the second experimental round are presented in Figure 6. The P300 response is superimposed on the deflections that are rhythmically evoked by the stimulus itself. The rhythm of these evoked potentials is transiently disturbed by the positive deflection. In condition C175, negative deflections appear to miss a cycle (see Figure 6, column 2). In condition C300, the P300 response has more time to develop which results in a positive going potential (see Figure 6, column 1). In condition C300s, most subjects show no markedly different traces for non-targets and targets. Subject VPzq showed very pronounced positive deflections for all conditions (including C300s) and was also the best scoring subject in most conditions of the BCI experiments. Scalp topographies from condition C175 (see Figure 7) are more diffuse and the class difference has shifted toward the frontal areas when compared to the longer ISI of condition C1000. This change is also visible when looking at the channels that were selected for feature extraction during the classification routine (Figure 8).

Negative deflections for the second experimental round are not discussed here, as the ROC values were low.

### Stimulus intensity

Before all sessions, subjects could adjust the speaker loudness for all directions to obtain a subjective equality in stimuli. The majority of subjects reported the preset speaker loudness (calibrated at 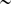
58 dB) to be perceptually equal. Therefore, only the three subjects that changed the loudness of at least one speaker are reported in Table 5. Subject VPig and VPzq (BCI) requested all speakers to be louder (about 3–5 dB) than initially set. In this case the initialization was not used by the subject to balance the speaker loudness, but to change the overall loudness. The classification results for VPzq in the BCI experiments were higher than average, with scores reaching 100% in most conditions.

**Table 5:** Speaker loudness.

Subject	Exp	Speaker location							
		1	2	3	4	5	6	7	8
VPzq	Phys.	-	-	-	60.8	60.2	59.7	-	-
VPig	Phys.	61.4	61.1	61.2	60.1	60.2	60.1	61.2	60.4
VPkj	BCI	-	56.9	56.2	x	x	X	-	-
VPzq	BCI	63.4	63.8	62.9	x	x	X	63.7	63.2

Speakers were calibrated to equal loudness (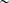
58 dB). Before each session, the subject could adjust this loudness for individual speakers to have subjectively equal loudness. Most subjects did not make changes; only those that did are reported here. Subjects are grouped according to experimental rounds. *Exp* refers to the experimental round. *Speaker location* refers to the speaker labels given in Figure 1. -  =  unchanged, x  =  unavailable. All values are in dB.

In the physiological experiments, VPzq adjusted only the three speakers in the back. Subject VPkj decreased the loudness of the speakers right and front-right. This is possibly to account for user specific hearing differences between ears or to counterbalance the perceptual damping of sound sources in the back.

### Key response result

The key-reponse task (condition Cr) was performed by subjects in the physiological experiments. Performance results can be found in Table 4. Error scores (in percentages) were calculated with equation 3. The number of true negatives is excluded from this equation, because its large number would mask the error size.
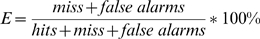
(3)


Although no subject showed a perfect score, number of errors was under 10% for all subjects but VPig. Subject VPig, with an error rate of 22.1%, was one of two subjects who reported to have difficulty with sound localization in natural settings. Subject VPip, who also reported this, had an excellent result with an error rate of 1.4%. Both received a practice round prior to the recordings and had a maximum selection score of over 90% in a preliminary classification test.

**Figure 9:**
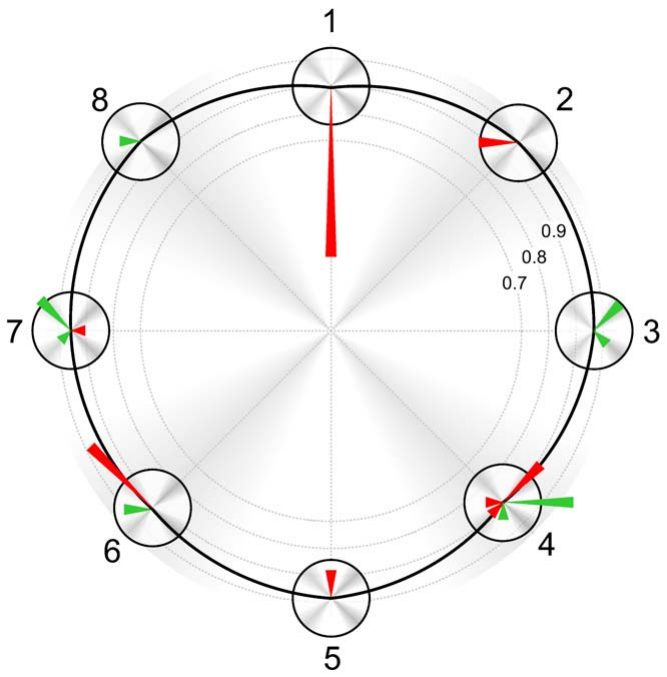
Polar sensitivity plot for condition Cr. The confusion matrices for condition Cr of all subjects are summed and represented as sensitivity plot. The black line indicates the sensitivity at each speaker location. Direction confusion is represented by the green (neighboring direction) and red (other) arrows. The length of the arrow indicates the amount of error in that direction. Speaker 1 (front) and 5 (back) are difficult to distinguish, as can be seen from their exclusive confusion. Direction labels correspond to those in Figure 1.

The grouped performances of subjects on different directions can be found in Table 6; the corresponding confusions in Figure 9. The first observation to be made is the confusion of the front speaker with the rear speaker. All eight false alarms on the front trials are due to confusion with the rear speaker. Vice versa, the only false alarm on the rear trials is a confusion with the front speaker. Several subjects also reported to have difficulty with this distinction. We therefore excluded the rear speakers from the BCI experiments.

**Table 6:** Averaged performance for different directions (condition C1000).

Direction	RT [ms]	Hits	False alarms	Misses	Error
Front	483 (168)	67	8	1	11.8%
Front-right	414 (121)	40	1	4	11.1%
Right	431 (126)	84	3	0	3.5%
Back-right	552 (211)	83	10	1	11.7%
Back	518 (242)	57	1	3	6.6%
Back-left	499 (184)	51	4	1	8.9%
Left	408 (100)	108	5	0	4.4%
Front-left	403 (121)	75	1	1	2.6%
**Average**	463.5 (159.1)	-	-	-	7.6%

Reaction times (standard deviation in parentheses) and errors for all subjects were averaged according to direction of target stimulus. Types of errors made can be found in Figure 9. Due to random target assigning, not all directions are designated as a target equally often. The longest reaction times are found in the rear speakers 3 speakers and the frontal 1.

Looking at the distribution of false alarms relative to the target cue, in total 14 false alarms are made on cues directly neighboring the target and 19 errors on the other cues. When normalizing for the amount of cues (two direct neighbors versus five others) this means that the probability of a false alarm on a neighboring cue (1.22%) is almost twice as high as the probability of a false alarm on any of the other directions (0.66%). When not taking the front and rear speaker into account, the difference between these increases (1.6% for neighboring cues and 0.45% for other cues).

### Channel selection

In every fold of the cross validation, the best set of 20 channels is chosen based on the ROC value. The distribution of selected channels for the different experimental settings can be found in Figure 8. As can be seen, channels that have been selected for their predictive power for the negative ROC values are consistently concentrated in the mid-frontal areas. The channels that were selected in condition C1000, during preliminary classification, for their predictive power for the positive ROC values are focally located over the parietal- and occipital area. This is consistent with the assumption that negative ROC values are associated with attentional differences of the eary negative waves [41] and positive ROC values are associated with the P300 wave differences [42]. It is also consistent with the ROC topographies in Figure 3 and 4. When the ISI is decreased in the BCI experiments, the negative channels are still concentrated around the *Fz* channel, whereas the distribution of the positive response becomes more diffuse.

### Classification

**Figure 10:**
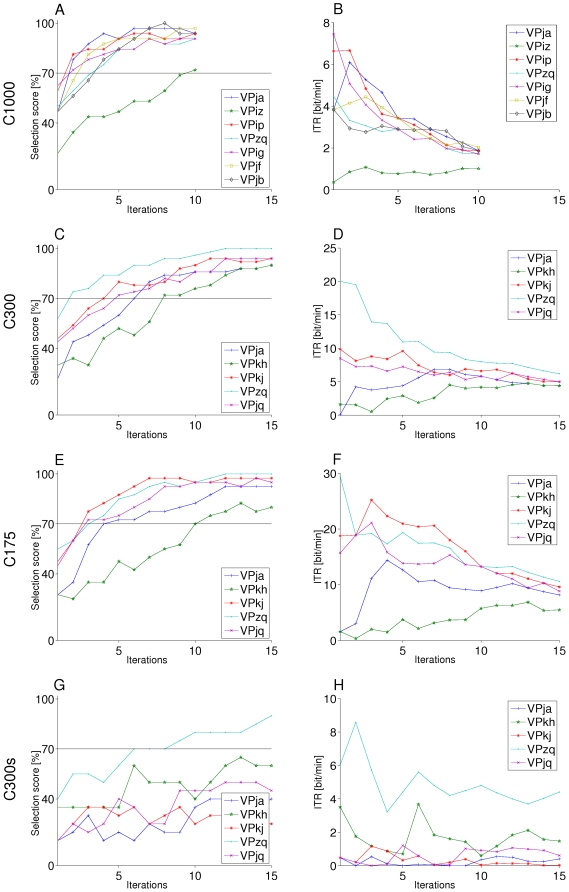
Subject performances for all conditions. The left column shows the selection scores and the right column shows the corresponding ITR. Both are plotted as a function of the number of iterations. Each rows indicates a different condition. The horizontal line in the left column indicates the 70% threshold. Although we do not report on the classification on condition C1000, we have included the figures here for comparison. They have only 10 iterations, as no more stimuli were presented in the physiological experimental round. Accuracy scores for condition C1000 increase faster for than for the other conditions. However, because of the long ISI (1000 ms) the ITR is relatively low.

Tables 7a–c give the classification- and selection results for the BCI experiments. When using a single iteration for finding the target direction, all subjects scored a selection accuracy below 70% in all conditions. When using multiple iterations the score for most subjects went up quickly for conditions C300 and C175. For control condition C300s, this increase could not be observed for most subjects (see Figure 10).

**Table 7:** Classification performance for all BCI conditions.

Subject	Classification [%]	Target score [%]	Selection [%]	70% Thresh.	Max. ITR 70%	Max. ITR 90%
VPja	69.81	62.12	90.00 (15)	6	6.86 (7)	4.41 (15)
VPkh	**74.27**	69.02	**90.00** (15)	8	4.78 (13)	4.41 (15)
VPkj	73.19	68.08	94.00 (11)	4	9.60 (5)	6.81 (11)
VPzq	78.74	74.89	**100.00** (12)	2	19.50 (2)	11.02 (6)
VPjq	74.54	69.10	94.00 (12)	5	7.25 (5)	6.25 (12)
**Mean**	74.11	68.64	93.60 (13.0)	5.0	9.60 (6.4)	6.58 (11.8)
			a) C300			
VPja	**72.20**	63.00	**92.50** (12)	4	14.41 (4)	10.21 (12)
VPkh	68.83	61.39	82.50 (13)	10	6.87 (13)	- (-)
VPkj	**77.48**	71.67	**97.50** (7)	3	25.20 (3)	20.60 (7)
VPzq	**79.89**	75.22	**100.00** (12)	3	19.36 (5)	17.51 (7)
VPjq	**75.66**	72.56	**97.50** (14)	3	21.10 (3)	15.32 (8)
**Mean**	74.81	68.76	94.00 (11.6)	4.6	17.39 (5.6)	15.91 (8.5)
			b) C175			
VPja	57.96	34.66	40.00 (11)	-	- (-)	- (-)
VPkh	63.35	47.11	65.00 (13)	-	- (-)	- (-)
VPkj	60.32	41.26	35.00 (3)	-	- (-)	- (-)
VPzq	72.20	59.79	90.00 (15)	6	5.60 (6)	4.41 (15)
VPjq	62.75	42.12	50.00 (12)	-	- (-)	- (-)
**Mean**	63.32	44.99	56.00 (10.8)	6.0	5.60 (6.0)	4.41 (15.0)
			c) C300s			

For explanation of the various conditions see the *Methods* section. *Classification (%)* refers to the binary classification score on the artifact free dataset, i.e. the correct classification of individual subtrials. *Target score (%)* is the same but only considering target subtrials. The difference between these is possibly due to unbalanced training set. *Max. selection* is the maximum selection score reached for each subject, *70% Thresh*. refers to the minimum number of iterations needed for averaging to obtain a 70% selection score. *Max. ITR (70%)* is the maximum ITR reached when considering only those numbers of averaging that resulted in a 70% selection score or higher. *Max. ITR (90%)* is the equivalent with only selection scores above 90%. Number in parentheses in *Max. selection*, *Max. ITR (70%)* and *Max. ITR (90%)* indicate the number of averages needed for the result. Bold numbers indicate the best result per subject over the three conditions. For most subjects, ITR values are highest for condition C175. See Figure 10 for results from more averaging steps and the corresponding ITR. See the *Analysis* subsection for definitions of classification- and selection score.

In condition C300 (see Table 7a), four out of five subjects reached a selection score of 70% or higher already after using six iterations. The fifth subject reached this threshold after eight iterations. One subject had a selection score of 100% when using 12 or more iterations, all other subjects eventually scored 90% or higher. The average maximum selection score was 93.6%. Average maximum ITR scores were 9.60 and 6.58 for the 70% and 90% constraint, respectively.

For condition C175 (see Table 7b), four out of five subjects had a selection score of 70% or higher when using four iterations. Subject VPkh only reached this threshold after using ten iterations. Subject VPzq reached a 100% selection score when using 12 iterations. All but subject VPkh eventually reached a 90% selection score with an average maximum score of 94.00%. Average maximum ITR scores were 17.39 and 15.91 for the 70% and 90% constraint, respectively.

In condition C300s, both the classification- and selection scores were lower (see Table 7c). Subject VPzq reached the 70% threshold already after using 6 iterations and had a maximum score of 90% on this control condition. The subject reported to regularly sing in a choir. For all other subjects, selection scores did not rise above 70%. Because condition C300s contained less subtrials (1500 versus 3500 and 3000 in the C300 and C175 condition respectively), it could be argued that the lower classification- and selection scores are due to the lower number of training samples. However, running conditions C300 and C175 with only 1500 subtrials resulted in similar scores as those currently reported. Any difference between conditions is thus due to information added by the spatial localization of the stimuli.

Cross validation was also performed on the data from the physiological experiments. Although both classification- and selection scores were comparable or better, we do not report on these results extensively here as the long ISI makes the system intrinsically slow. For comparison, Figure 10 does show these results. Note that the bad score for subject VPiz is due to the removal of over 50% of the trials.

For all conditions and subjects, the classification score of target stimuli is lower than the overall classification score (see column ‘Target score’ in Table 7). The classifier favors the decision towards the non-targets, which is due to the bias that exists in the training set. Balancing of the training set might increase the classification score, but has not been applied here.

## Discussion

We discuss here a new experimental paradigm for an auditory BCI. In contrast to most other auditory BCI setups, our setup involves an intuitive multi-class paradigm that can readily vary in the number of classes. So far, it has only been tested offline and on healthy subjects. The results show that all subjects were able to reach a selection score over 70% in the conditions with spatial cues (C300 and C175). Actually, all but one subject reached selections scores higher than 90%. Performance on the control task was for all but one subject below the 70% threshold, showing that the spatial location adds vital information to the cue.

As can be seen in Figure 10, the increase in selection score is highest for condition C1000. The P300 waveform in this condition has time to reach a peak and recover to baseline to some extent and is more typical. For the other conditions, more iterations were necessary for a selection score above 70% i.e., the slope is less steep. Here, the P300 is no longer the clear and pronounced positive peak, as responses evoked by new stimuli disturb the potential buildup. ROC values were generally larger for the C1000 condition as compared to the faster conditions.

For the BCI experiments, especially C175, the area with the highest ROC values had shifted to a more frontal position when compared to condition C1000. Generally, the latency of the P300 wave is shorter over frontal electrodes [17]. Thus, the positive deflection starts developing over the frontal electrodes and moves back. One could therefore explain this shift by an interruption of the developing P300 by the potentials evoked by the presentation of the next stimulus. However, latency differences between frontal and parietal areas are in the range of milliseconds [43], which makes it an unlikely explanation. A variant of the P300, the novelty P300 and P3a, generally starts more anterior than the classic P300 [44]. The fact that it is present after an unknown stimulus and habituates quickly also makes it an unlikely candidate. Neurophysiological P300 research mostly uses longer ISI to avoid the overlay of multiple stimuli. In BCI research, where short ISI is common practice, scalp topographies are not often reported. The reason for the shift of the P300 to the front therefore remains unclear.

One measure of reporting the usefulness of a BCI is the ITR. It depends on the selection accuracy, the time necessary for a choice and the amount of classes. Because of the long ISI in condition C1000, the ITR is inevitably low. However, with the pronounce P300 response it functioned as a proof of concept. In an auditory BCI setup, the spatial properties of the cue by itself can be enough to consistently elicit a classifiable P300 response.

With an ISI that is almost six times shorter in condition C175, extra iterations can still produce an overall fast BCI. Also, some subjects reported the faster ISI to help them focus on the task at hand. With successful classification in trials with an ISI as short as 175 ms, the maximum ITR reached an average of 17.39 bits/minute for five subjects (best subject 25.20), considering only 70% correct selection scores. Kanoh et al. [7] reported an average ITR of around 5 bits/minute on their binary BCI, but only when they used all data for training and testing, thereby applying the classifier to data it had already seen. In another binary auditory setup [2], an ITR of between 4 and 7 bits/minute is reported. Our system owes its high ITR to its genuinely multi class nature. Another multi class, auditory BCI is reported on before [5]. It used spoken numbers as the stimulus for eliciting an ERP and an average ITR of 1.48 bits/minute was reported for their online approach. This could be improved to 4.66 bits/minute when they determined the individual optimal number of iterations in an offline analysis. Recently, [10] reported on their multi-class auditory BCI with a maximum online ITR of 5.64 bits/minute in the auditory only condition. The average ITR remained relatively stable over the different sessions.

Visual P300 BCI systems are known for their fast operation and corresponding high ITR. In a recent online visual speller study [21], average ITR values of 32.15 bits/minute were reported. For this they used four subtrials with an average classification score of over 80%. Maximum ITR for a single subject was as high as 92.32 bits/minute using two subtrials. It can be assumed that the average ITR will further increase, when the optimal number of subtrials is determined for each subject individually. Even in the original application of the visual spelling system in 1988 [20], ITR values of 12.0 bits/minute (or 10.68 bits/minute according to equation 2) were reported. For a comparison of ITR of several BCI systems, see [45]. It is thus clear that auditory BCI systems lag behind in their performance. The setup proposed here takes a step in closing this gap between visual and auditory performance.

The average ITR for condition C175 went down to 15.91 bits/minute (best subject 20.60 bits/minute) when only 90% correct selection scores were considered. Although this is a drop in ITR of about 9%, it is a score that is still competitive with other auditory BCI systems and has a much higher accuracy barrier. This high accuracy and corresponding ITR encourage the further development of this paradigm. This could for instance be achieved by using a multi class classifier [46], instead of the binary classifier used.

Performance in condition C300s was low, with only one out of five subjects crossing the 70% threshold. Possibly, the performance in the control condition can be improved when the cues differ more in their physical properties i.e., if the difference in pitch is larger or natural sounds are used as in [10]. This would make distinguishing the targets from the non-targets easier and thus, maybe, an auditory multi-class BCI could also be based on this single speaker setup. However, remembering a pitch is rather difficult for some subjects, whereas recognition of a spatial direction is automatic. Subject VPzq actually reached a selection score of 90% in condition C300s. As this subject reported to regularly sing in a choir it could be hypothesized that for him the task was easier to perform and therefore still elicited a P300 response.

Currently the stimuli are presented in free-field i.e., with a dedicated speaker for every direction. Initial tests with stimulus presentation over in-ear headphones showed that accurately identifying the target direction was difficult. However, we believe that by using the complex cue from the BCI experiments and more advanced methods for creating virtual 3D audio, it will be possible to reduce the large setup to stereo ear phones.

As shown by the polar sensitivity plot of the key response task, there is a higher chance of mistaking a target with one of its direct neighbors than other stimuli. Possibly, these neighboring directions fall within the attentional gradient [28], [29]. Also, it seems that more trials are misclassified in the rear than in other directions. It was shown that the spatial resolution of hearing is higher in the frontal region than toward the sides in [41]. Their experiments were on the front-right quadrant only. However, it shows that a more informed placement of the speakers around the subject might improve their ability to distinguish the different cues.

A wide range of applications is possible as the directions can be mapped to any choice and the number of directions is flexible. One area in which our method might prove useful is auditory BCI based on spoken words [5]. A BCI with spoken word input might prove an intuitive alternative to the somewhat unnatural tones. However, it introduces problems such as increased latency jitter in the P300 onset which may hinder the classification. Spoken words that contain spatial information might lead to a more pronounced response because it is easier to focus on the direction. Also, there is no longer the need to hear a large part of the word before actual recognition can take place. Recognition is then based on the spatial location, whereas the spoken word functions as a reminder of which cue is mapped to a certain direction. Similarly, the paradigm described in [10] might benefit from adding spatial information to the cues.

However good the offline results are, they will need to be confirmed in an online setting. Preparations for an online study are currently undertaken.

## Supporting Information

File S1 Cues as used in the BCI experiments. Cues consist of bandpass filtered noise with a tone overlay. See Table 1 for their properties.
(0.02 MB ZIP)
